# Can psychosocial and socio-demographic questions help identify sexual risk among heterosexually-active women of reproductive age? Evidence from Britain’s third National Survey of Sexual Attitudes and Lifestyles (Natsal-3)

**DOI:** 10.1186/s12889-016-3918-8

**Published:** 2017-01-04

**Authors:** Natalie Edelman, Jackie A. Cassell, Richard de Visser, Philip Prah, Catherine H. Mercer

**Affiliations:** 1 Brighton & Sussex Medical School, 318b Mayfield House, Village Way, Brighton, Falmer BN1 9PH UK; 2 University of Brighton, Brighton, UK; 3 School of Psychology, Room 1C12 Pevensey1, University of Sussex, Brighton, BN1 9RH UK; 4 Centre for Sexual Health and HIV Research, University College London, 3rd Floor Mortimer Market Centre off Capper Street, London, WC1E 6JB UK

**Keywords:** Sexual risk, Survey, Psychosocial, Socio-demographic, Women

## Abstract

**Background:**

Contraceptive advice and supply (CAS) and sexually transmitted infection (STI) testing are increasingly provided in primary care. Most risk assessment tools are based on sexual risk behaviours and socio-demographics, for use online or in specialist services. Combining socio-demographic and psychosocial questions (e.g. religious belief and formative experience) may generate an acceptable tool for targeting women in primary care who would benefit from intervention. We aimed to identify psychosocial and socio-demographic factors associated with reporting key sexual risk behaviours among women in the British general population.

**Methods:**

We undertook complex survey analysis of data from 4911 hetero-sexually active women aged 16–44 years, who participated in Britain’s third National Survey of Sexual Attitudes and Lifestyles (Natsal-3), a national probability sample survey undertaken 2010–2012. We used multivariable regression to examine associations between the available psychosocial and socio-demographic variables in Natsal-3 and reports of three key sexual behaviours: a) 2+ partners in the last year (2PP); b) non-use of condoms with 2+ partners in the last year (2PPNC); c) non-use of condoms at first sex with most recent sexual partner (FSNC). We adjusted for key socio-demographic factors: age, ethnicity and socio-economic status (measured by housing tenure).

**Results:**

Weekly binge drinking (6+ units on one occasion), and first sex before age 16 were each positively associated with all three sexual behaviours after adjustment. Current relationship status, reporting drug use (ever), younger age and living in rented accommodation were also associated with 2+ partners and 2 + partners without condoms after adjustment. Currently being a smoker, older age and respondent ethnicity were associated with FSNC after adjustment for all other variables. Current smoking status, treatment for depression (last year), and living at home with both parents until the age of 14 were each associated with one or more of the behaviours.

**Conclusions:**

Reported weekly binge drinking, early sexual debut, and age group may help target STI testing and/or CAS among women. Further research is needed to examine the proportion of sexual risk explained by these factors, the acceptability of these questions to women in primary care and the need to customise them for community and other settings.

**Electronic supplementary material:**

The online version of this article (doi:10.1186/s12889-016-3918-8) contains supplementary material, which is available to authorized users.

## Background

Much sexual health research to date has focused on how sexual behaviour is associated with acquisition and transmission of sexually transmitted infection (STI), and - to a lesser degree- with unplanned pregnancy (UP) [1]. These approaches have supported the identification of higher risk populations (such as young people and Men who have Sex with Men), and the targeting of these populations with sexual health interventions, e.g. England’s National Chlamydia Screening Programme [2]. Accordingly, a number of sexual risk assessment tools have been developed for clinical use. Although none of these address risk of UP, several have been developed to identify those at risk of STIs, typically based on sexual behaviour and socio-demographic items such as age, ethnicity, gender and sexual orientation [3, 4].

There is also growing evidence that ‘psychosocial’ factors such as relationship qualities [5, 6], mental health [7], and substance use [8] may also be associated with adverse sexual health outcomes, and with the sexual risk behaviours which mediate them. Thus, they may be of use in identifying at-risk individuals for targeted intervention. Within social epidemiology, psychosocial factors have been defined as those which influence health outcomes by affecting biology, behaviour or psychology [9]. For the purposes of this study we define it as factors which do not fit within the categories of socio-demographics, sexual behaviour or health psychology constructs such as risk perception and self-efficacy. Previous use of this definition identified factors that concern health, substance use, formative experiences, lifestyle, and relationships [10].

This exploratory study was undertaken to underpin the development of a psychosocial clinical prediction rule (CPR) [11], being developed primarily for use in targeting STI testing, safe sex advice and contraception advice and supply (CAS) to women of reproductive age attending British primary care settings. As such, this study reflects a biomedical understanding of women’s sexual health risk, contrasting with the broader definition of sexual health endorsed by the World Health Organization [12]. CPRs are tools used in clinical assessment to direct the nature of clinical intervention, based on response to a short set of patient questions. The CPR which this work underpins is envisaged as a brief questionnaire which women can self-complete in order to assess their need for STI testing and/or CAS. In this exploratory study we hypothesised that combining psychosocial and socio-demographic factors may help explain variance in sexual risk behaviour within populations, enabling targeting of those at greater risk of adverse sexual health outcomes without the need for clinical staff to take a sexual history. This may be advantageous as sexual history-taking can be time-consuming for staff and patient; and may be perceived as intrusive where the patient has attended primary care for non-sexual health matters. This approach may also flag up psychosocial issues, such as binge-drinking, which warrant treatment in their own right and may also be antecedents of a broader range of sexual risk – such as risk of STIs through partners, and inconsistent contraception use.

Therefore our aim was to explore the extent to which psychosocial and socio-demographic factors might be used to identify women experiencing higher levels of sexual risk. As a proxy, in this study we examined hypothesised associations between psychosocial and socio-demographic factors with key sexual risk behaviours, using data from a national probability sample survey.

To this end we address three research questions:

1. Which psychosocial and socio-demographic factors are associated with key sexual risk behaviours?
2. Which psychosocial and socio-demographic factors, if any, are associated across different sexual risk behaviours?
3. Do observed associations between psychosocial factors and sexual risk behaviours remain after adjustment for key socio-demographic factors: age group, ethnicity and socio-economic status (measured by housing tenure)?

## Methods

Data from the third National Survey of Sexual Attitudes and Lifestyles (Natsal-3) were analysed. This probability sample survey of the British resident population aged 16–74 years was conducted from 2010 to 2012 inclusive, using a multi-stage, clustered and stratified sampling methodology. In line with standard practice for UK surveys, and in response to evidence suggesting that signing a consent form might lead to a greater sense of obligation to complete the interview, we obtained verbal rather than written consent. Full details of the study design are described elsewhere [13, 14]***.*** A sample size of 15,162 was achieved - an overall response rate of 57.7% with only very small (typically 1–3%) amounts of item non-response [13]. Natsal-3 asked about subjects, including sexual partners and practices, experience of depression, substance use, STI diagnosis, unplanned pregnancy, sexual function [15] and non-volitional sex [16]***.*** This has permitted investigation of the wider social and health contexts of sexual health and behaviour [17].

The Natsal-3 dataset provides an opportunity to examine the existence of associations in the general population; which, being heterogeneous in sexual risk is broadly representative of a primary care clinic population. This contrasts with specialist sexual health clinic populations which tend to report greater sexual risk behaviour [18]. The analyses presented in this paper were conducted on a subset of Natsal-3 respondents, defined as women aged 16–44 years who reported heterosexual sex (defined as anal, vaginal, or oral sex with a male partner) in the last year. These criteria were used to generate findings most applicable to the development of the CPR.

### Outcome measures

Low population prevalence of STI diagnosis, abortion and unplanned pregnancy in the last year, found in this Natsal-3 sample, meant that there was insufficient statistical power to analyse these as outcomes in this study. Instead three sexual risk behaviour measures [19] were selected as proxies for these adverse sexual health outcomes, on the grounds that STI screening and CAS may be offered in response to reported sexual risk behaviour.

To minimise the risk of Type II error in the analysis we selected three key sexual risk behaviour variables which were reported by more than 10% of the sub-population of interest, equating to *n* ≥ 500 women. Having 2+ partners in the last year (abbreviated here as 2PP) was chosen as a variable known to be associated with STI acquisition and commonly used for clinical and research purposes [20, 21]. Non-use of condoms with 2+ partners in the last year (abbreviated here as 2PPNC) was chosen as it combines multiple partnerships and condom use to provide a more precise indicator of STI risk behaviour. Specifically this variable refers to at least one episode of non-use of condoms, occurring with two or more partners. Finally, we selected non-use of condoms at first sex with most recent partner (including those only reporting having sex once with their most recent partner), abbreviated here as FSNC. This variable represents a sexual encounter when ‘risk’ of infection or unplanned pregnancy might be most highly perceived and we might therefore anticipate a greater likelihood of condom use. However, as a single sexual encounter is unlikely to result in significant risk, this exploratory variable constitutes a potential proxy indicator of broader sexual risk experiences. By focusing only on non-use of condoms this variable may also be more representative of those at risk of STIs through partner, rather than own, sexual risk behaviours.

### Psychosocial and socio-demographic variables

As this study was undertaken to support the development of a CPR, investigation focused on identifying socio-demographic and psychosocial items strongly and commonly associated with measures of sexual risk, which would also be brief, acceptable and easy to score as clinical questionnaire items.

Most of the psychosocial variables selected for testing in bivariate analysis fitted into one of the following broad topics: substance use, mental health, general health, sexual orientation, formative experiences involving family, formative experiences involving sex, partner descriptors and relationship status and satisfaction. We chose a measure of sexual orientation that did not incorporate sexual behaviour. This was driven by a decision to avoid incorporating sexual behaviour items as exposures, coupled with a concern that sexual orientation items which are defined by sexual behaviour with men and women, may be confounded by multiple partnerships.

Questions asked in Natsal-3 regarding non-volitional sex [16] were deemed unlikely to be acceptable for use in a CPR and were therefore excluded from our analyses. Childhood sexual abuse (CSA) was however included covertly, within a dichotomous variable constructed for first heterosexual intercourse <16 years (yes/no), as there is evidence of correlations between sexual risk and morbidity, CSA and early sexual debut [22]. This contrasts with other Natsal-3 papers which report on first heterosexual intercourse <16 years, but exclude from their analysis those reporting first sex under the age of 13 years [23, 24].

Where more than one brief and easy-to-score psychosocial variable remained within the same topic and each was found to be associated with the outcomes of interest, the variable with the highest frequency of response was selected for multivariable analysis. This approach was founded on the rationale that rarer psychosocial factors will explain a low proportion of variance in sexual risk behaviour (no matter how strong the association) and will therefore have less utility in the general population. This corresponds with the notion of ‘adequate prediction’ [11].

Forty-two variables were initially identified from the Natsal-3 dataset as being representative of psychosocial factors (see Additional file 1). The original questions from which they were derived can be viewed at http://www.natsal.ac.uk/natsal-3/core-survey/questionnaire.aspx. Of the 42 variables identified, ten were selected by applying the approach described above. These were:

- relationship status (recoded as cohabiting with partner, stable relationship not cohabiting, not in a relationship but previously cohabited, not in a relationship and never cohabited)
- sexual identity (recoded as heterosexual/not heterosexual)
- belong to any religion now (coded as yes/no)
- smokes cigarettes nowadays (recoded as yes/no)
- weekly drinking of 6+ units of alcohol on one occasion (recoded as yes/no)
- ever taken non-prescribed drugs (yes/no)
- received treatment for depression in the last year (recoded as yes/no)
- lived with both parents until the age of 14 (yes/no)
- first heterosexual intercourse before the age of 16 years (yes/no)
- most recent partner’s ethnicity (white/Asian British/black British/other)

There is strong existing evidence for associations between sexual morbidity and socio-demographic factors [25, 26]. Therefore age group [15], ethnicity and socio-economic status were also assessed for model inclusion, using rental of current home as a brief and acceptable proxy indicator of the latter.

### Statistical methods

In order to make the sample broadly representative of the target population according to the 2011 Census, selection probability weights were applied to the data to adjust for the unequal probability of selection and then post-stratification weights were applied to adjust for non-response bias [14].

Aggregated variable categories were used only where it was necessary to boost cell frequencies or render items briefer and easier to complete. Aggregation was based only on observed gaps in continuous data or on overlapping confidence intervals for categorical data - with the exception of body weight which was aggregated using the weight boundaries for body mass index (BMI) on a typical 5’7” woman. This approach reduced the number of bivariate analyses and therefore of Type 1 error. Hence, we did not apply a Bonferroni correction to our analyses [27].

Bivariate analyses were conducted with each psychosocial and socio-demographic variable described above, for each of the three sexual risk behaviour variables. Those which demonstrated an association at *p* ≤ 0.05 were entered into a multivariable model for that behaviour. Psychosocial variable associations between 0.05 ≤ *p* < 0.10 (after adjustment for socio-demographic variables) were also entered. This approach provided consistency with criteria for removal of variables from the model which was set at *p* ≥ 0.1. Backwards stepwise multivariable logistic regression was used for the chosen psychosocial and socio-demographic variables to identify which combination of these were retained in the model using pre-set criteria. This approach was chosen as many concerns with this type of analysis were reduced in this study. I.e. we selected empirically only a modest number of variables, and risk of Type II error was reduced by the large sample size and by setting the criteria for removal of the model at *p* ≥ 0.1. This criterion, and the backwards elimination approach, also acted to reduce suppressor effects [37]. Table 1 in the Results section presents the percentage reporting that risk behaviour in each category of each exposure, the crude odds ratios for each category of that exposure, and the global P value for each exposure. For each of the exposures entered into the multivariable model, Table 1 also presents the global P value, with adjusted odds ratios for each exposure that was retained after adjustment for all other variables.

**Table 1:** Psychosocial and socio-demographic characteristics of sample and their association with three key sexual risk behaviours

		Outcomes:									
	% of sample (weighted) reporting characteristic	2+ sexual partners in the last year (2PP)			2+ sexual partners in the last year without condom use (2PPNC)			Non-use of condoms at 1^st^ sex with most recent partner (FSNC)			Denominators^c^ (unweighted, weighted)
Psychosocial/socio-demographic factor:		% reporting outcome	Crude OR (95% CI)	Adjusted^d^ OR (95% CI)	% reporting outcome	Crude OR (95% CI)	Adjusted^d^ OR (95% CI)	% reporting outcome	Crude OR (95% CI)	Adjusted^d^ OR (95% CI)	4911,3431
Relationship status			*p* < 0.0001	*p* < 0.0001		*p* < 0.0001	*p* < 0.0001		*p* = 0.355	*p* = 0.7458	
Cohabiting with partner	64.9% (63.4–66.4)	5.6%	1.00	1.00	2.7%	1.00	1.00	41.1%	1.00	1.00	2635,2226
Stable relationship not cohabiting	17.1% (16.0–18.2)	30.9%	7.73 (6.09–9.82)	5.18 (3.96–6.78)	17.7%	8.98 (6.30–12.8)	6.37 (1.33–9.37)	37.0%	0.78 (0.65–0.93)	Not retained in model	1128,585
Not in relationship but has cohabited	8.6% (7.9–9.5)	49.8%	17.3 (12.9–22.9)	13.3 (9.81–18.1)	30.4%	17.0 (11.7–24.6)	13.4 (8.88–20.2)	48.0%	1.30 (1.04–1.62)		552,296
Not in relationship & never cohabited	9.4% (8.5–10.3)	53.4%	21.1 (15.8–28.2)	14.2 (10.4–19.2)	29.2%	17.8 (12.4–25.6)	13.5 (8.94–20.5)	36.7%	0.81 (0.64–1.01)		569,321
Sexual identity			*p* = 0.002	*p* = 0.7041		*p* = 0.16	Not entered into model		*p* = 0.673	Not entered into model	
Heterosexual	97.7% (97.2–98.2)	21.8%	2.12 (1.33–3.38)	Not retained in model	12.3%	1.00		40.4%	0.91 (0.59–1.41)		4781,3348
Other	2.23% (1.8–2.81	33.1%	1.00		17.4%	1.54 (0.84–2.79)		40.2%	1.00		121, 78
Currently a smoker			*p* < 0.0001	*p* = 0.013		p < 0.0001	*p* = 0.5628		*p* < 0.0001	*p* = 0.002	
Yes	28.3% (27.0–29.8)	31.7%	2.25 (1.91–2.65)	1.32 (1.06–1.63)	18.9%	2.13 (1.73–2.61)	Not retained in model	46.2%	1.31 (1.13–1.52)	1.31 (1.10–1.55)	1560,972
No	71.7% (70.2–73.0)	17.5%	1.00	1.00	9.4%	1.00		37.7%	1.00	1.00	3351,2459
Usually binge-drink at least weekly			*p* < 0.0001	*p* < 0.0001		*p* < 0.0001	*p* < 0.0001		*p* < 0.003	*p = 0.004*	
Yes	13.9% (12.8–15.2)	40.6%	2.91 (2.34–3.67)	2.11 (1.63–2.73)	26.5%	3.05 (2.37–3.93)	2.00 (1.51–2.64)	47.8%	1.35 (1.11*–*1.66)	*1.36 (1.10–1.68)*	643,428
No	86.1% (84.8–87.2)	19.5%	1.00	1.00	10.6%	1.00	1.00	39.7%	1.00	*1.00*	3748,2643
Ever used non-prescribed drugs			*p* < 0.0001	*p* = 0.012		*p* < 0.0001	*p* = 0.018		*p* = 0.274	*p* = 0.6288***	
Yes	39.4% (37.7–41.0)	28.3%	1.88 (1.61–2.20)	1.31 (1.06–1.61)	17.1%	2.00 (1.62–2.48)	1.34 (1.05–1.71)	44.0%	1.08 (0.94–1.25)	Not retained in model	2875,1347
No	60.6% (59.0-62.3)	17.6%	1.00		9.0%	1.00	1.00	37.8%	1.00		2024, 2075
First heterosexual intercourse at <16 years of age^a^			*p* < 0.0001	*p* < 0.0001		*p* < 0.0001	*p* < 0.0001		*p* = 0.003	*p* < 0.0001	
Yes	50.2% (48.6–51.9)	27.9%	2.21 (1.86–2.63)	1.58 (1.26–1.96)	16.6%	2.66 (2.09–3.38)	2.00 (1.53–2.63)	43.0%	1.23 (1.07–1.41)	1.45 (1.23–1.70)	2756,1724
No	49.8% (48.1–21.4)	14.6%	1.00	1.00	6.9%	1.00	1.00	37.0%	1.00	1.00	2155,1707
Didn’t live with both natural (birth) parents to age 14			*p* < 0.0001	*p* = 0.1283		*p* = 0.013	*p* = 0.3855		*p* = 0.201^b^	*p* = 0.079	
Yes	73.2% (71.8–74.5)	26.8%	1.64 (1.40–1.91)	Not retained in model	11.4%	1.31 (1.06–1.62)	Not retained in model	39.7%	1.10 (0.95–1.28)	1.16 (0.98–1.37)	1516,920
No	26.8% (25.5–28.2)	19.9%	1.00		14.6%	1.00		42.0%	1.00	1.00	3395,2511
Received treatment for depression in last 12 months			*p* = 0.0002	*p* = 0.3394		*p* < 0.0001	*p* = 0.07		*p* = 0.012	*p* = 0.2312	
Yes	12.7% (11.6–13.8)	27.3%	1.55 (1.23–1.95)	Not retained in model	17.8%	1.75 (1.32–2.32)	1.39 (0.97–1.99)	48.2%	1.3 (1.06–1.59)	Not retained in model	662,435
No	87.3% (86.2–88.4)	21.2%	1.00		11.6%	1.00	1.00	39.2%	1.0		4247,2994
Belong to any religion now			*p* < 0.0001	*p* = 0.057		*p* < 0.0001	*p* = 0.4272		*p* = 0.169	Not entered into model	
Yes	46.3% (44.6–48.0)	17.7%	1.70 (1.44–2.01)	1.23 (0.99–1.51)	10.3%	1.52 (1.23–1.88)	Not retained in model	41.6%	0.91 (0.79–1.04)		2107,1584
No	53.8% (52.1–55.4)	25.3%	1.00	1.00	14.0%	1.00		39.5%	1.00		2796,1842
Most recent partner’s ethnicity			*p* < 0.0001	*p* = 0.1496		*p* = 0.016	*p* = 0.3926		*p* = 0.085	Not entered into model****	
White	86.1% (84.8–87.3)	21.6%	1.00	Not retained in model	12.3%	1.00	Not retained in model	39.5%	1.00		4248,2951
Asian British	5.6% (4.8–6.6)	11.7%	0.53 (0.34–0.83)		6.8%	0.63 (0.34–1.17)		55.6%	2.06 (1.54–2.76)		240,193
Black British	4.5% (3.8–5.3)	30.8%	1.61 (1.14–2.28)		14.1%	1.30 (0.82–2.06)		42.1%	1.10 (0.79–1.53)		221,152
Other	3.81% (3.2–4.5)	33.3%	2.09 (1.46–3.00)		18.8%	1.95 (1.26–3.03)		39.6%	1.07 (0.75–1.54)		195,130
Age group			*p* < 0.0001	*p* < 0.0001		*p* < 0.0001	*p* < 0.0001		*p* < 0.0001	*p* < 0.0001	
16–24 years	26.8% (25.5–28.1)	36.6%	1.00	1.00	20.3%	1.00	*1.00*	35.4%	1.00	1.00	1657,918
25–34 years	36.0% (34.5–37.5)	16.2%	0.30(0.25–0.36)	0.62 (0.50–0.78)	9.5%	0.39 (0.31–0.48)	*0.84 (0.63*–*1.12)*	39.8%	1.22 (1.05–1.43)	1.31 (1.11–1.55)	2215,1235
35–44 years	37.3% (35.6–39.0)	11.4%	0.18 (0.14–0.24)	0.55 (0.40–0.76)	5.7%	0.18 (0.13–0.26)	*0.53 (0.34*–*0.81)*	49.6%	1.89 (1.58–2.26)	2.22 (1.81–2.72)	1039,1278
Ethnicity			*p* < 0.0001	*p* = 0.987		*p* = 0.517	*p* = 0.9339		*p* = 0.019	*p* < 0.0001	
White	86.7% (85.4–87.8)	22.0%	1.00	Not retained in model	12.6%	1.00	Not retained in model	39.3%	1.00	1.00	4304,2969
Asian British	5.8% (5.01–6.8)	11.7%	0.49 (0.31–0.78)		5.0%	0.39 (0.19–0.78)		56.6%	2.10 (1.57–2.81)	2.89 (2.04–4.08)	239,200
Black British	3.6% (3.0-4.3)	29.9%	1.45 (0.97-2.17)		14.2%	1.12 (0.65-1.94)		47.7%	1.38 (0.94-2.03)	1.10 (2.61)	157,122
Other	3.9% (3.4–4.6)	27.7%	1.65 (1.12–2.42)		14.3%	1.44 (0.86–2.44)		38.6%	1.03 (0.72–1.49)	0.83 (1.80)	202,135
Currently renting home			*p* < 0.0001	*p* = 0.011		*p* < 0.0001	*p* = 0.09		*p* = 0.325^b^	*p* = 0.077	
Yes	47.6% (45.9–49.4	26.4%	2.10 (1.78–2.49)	1.31 (1.06–1.60)	15.2%	2.18 (1.76–2.71)	1.24 (0.97–1.59)	42.2%	1.07 (0.93–1.23)	1.15 (0.98–1.35)	2625,1626
No	52.4% (50.6–54.2)	16.9%	1.00	1.00	9.1%	1.00	1.00	38.2%	1.00		2261,1790

***Entered into model as significant effect found (*p* = 0.007) after controlling for age group and ethnicity

****Not entered into model as non-sig (*p* = 0.884) after controlling for ethnicity of respondent

^a^Including those who reported experiencing first heterosexual intercourse <13 years of age

^b^Statistically significant interaction between not living with both birth parents to age 14 and housing tenure

^c^Totals vary due to small frequencies of missing data

^d^Adjusted for all other variables in the model

## Results

This analysis included 4911 women aged 16–44 years who reported having heterosexual sex in the year prior to their Natsal-3 interview. Sample characteristics are given in Table 1 which also presents the model for each of the sexual behaviours analysed.

Having 2+ partners in the last year (2PP) was reported by 17.9% of respondents. Non-use of condoms with 2+ partners in the last year (2PPNC) was reported by 9.8% of respondents. Non-use of condoms at first sex with most recent partner (including those only have sex once with their most recent partner (FSNC) was reported by 41.5% of respondents. FSNC showed only small overlap with the other two behaviours - among these 41.5% of respondents, 8.5% reported 2PP in the last year and 6.3% reported 2PPNC in the last year.

First heterosexual intercourse at age <16 years and weekly binge drinking in the last year were each associated with all three sexual risk behaviours after adjustment for both socio-demographic variables and all other psychosocial variables, with adjusted odds ratios (AOR) ranging from 1.16 to 14.16. In contrast, two psychosocial variables - sexual identity and most recent sexual partner’s ethnicity - were not associated with any of the three sexual behaviours in multivariable regression.

Of the socio-demographic factors analysed, not owning a property was associated with both 2PP and 2PPNC (*p* < 0.05) after adjustment for all other variables, but only at (*p* = 0.077) with FSNC. Younger age group was positively associated with both 2PP and 2PPNC (adjusted), although 2PPNC showed no association between those aged 16–24 versus those aged 25–34 years. In contrast, younger women were *less* likely to report FSNC. After adjustment respondent ethnicity was associated only with FSNC.

Although current smoking and currently belonging to a religion were retained in the model for 2PP they were not retained in the model for 2PPNC. Conversely, treatment for depression in the last year was retained in the model for 2PPNC, but not in the model for 2PP. Not cohabiting with a partner was retained in the models for both 2PP and 2PPNC, but not the model for FSNC. In contrast, the variable for not living with both parents until age 14 was retained in the model for FSNC but not in the models for 2PP or 2PPNC. Although demonstrating a small effect size this exposure was reported by 26.8% of respondents.

Compared to currently cohabiting with a partner, each of the non-cohabiting response options showed very large effect sizes (odds ratios) in the 2PP and 2PPNC models, alongside high prevalence - not cohabiting but in a stable relationship (17%) and not being in a stable relationship (cumulatively 18%). For the 2PP and 2PPNC models there was little overlap between ‘stable relationship not cohabiting’ and the two ‘not in a relationship’ response options but great overlap between the latter two options, which also showed the greatest magnitude of effect. In the 2PP and 2PPNC models, drug use ever and currently renting showed very modest, though significant, effect sizes. In the model for FSNC largest effect sizes were observed for Black and Asian ethnicity, and for older age group (25–34 years versus 16–24 years, and 35–44 years versus 16–24 years).

## Discussion

Reporting weekly binge drinking in the last year, early sexual debut, younger age group and living in rented accommodation showed association with all three of the sexual risk behaviours studied (2PP, 2PPNC and FSNC). Notably, younger age was positively associated with multiple partnerships but negatively associated with FSNC. FSNC also showed quite different patterns of association to 2PP and 2PPNC overall, with much smaller effect sizes. Not living with both parents to the age of 14 years was associated with FSNC after adjustment for other factors, but not with 2PP or 2PPNC after adjustment. Not cohabiting with a partner was associated with 2PP and with 2PPNC after adjustment but not with FSNC. This may reflect that FSNC is not a good proxy for recent sexual risk, particularly as this variable was not limited to episodes of first sex occurring within the last year.

Binge drinking, early sexual debut and younger age have also been found to correlate with sexual risk in other population studies of sexual risk among women [10, 23, 28, 29]. However, observed associations between the dichotomous housing tenure variable and sexual risk contrast with previous Natsal-3 analyses which used comprehensive socio-economic variables [13]. Sexual identity was not found to be associated with sexual risk in our analysis. This may reflect one or more of the following: insufficient power to detect a significant association, a focus on heterosexual risk behaviour in defining the population of interest, or use of a sexual orientation measure based on identity rather than behaviour. However, on this latter point, two population surveys using non-behavioural measures of sexual identity have shown differences in partner numbers among adolescent women [30, 31].

Although a number of studies have examined associations between specific psychosocial factors and sexual risk behaviour, this study is unique in examining three sexual risk behaviours using a combination of psychosocial and socio-demographic items, in order to develop a CPR. Our findings are unique in demonstrating that binge drinking, early sexual debut, younger age and housing tenure remain significant when represented by briefly-worded and common variables, and might in principle be used to target sexual health interventions in primary care settings. This contrasts with existing sexual risk tools in two ways. Firstly, none of the existing tools have been developed for use using a population survey dataset. This reflects that only one has been developed for use in primary care (specifically for identifying STIs among paediatric primary care attenders in the United States [32]). Secondly existing tools focus primarily on sexual behaviour, symptoms and socio-demographics [33–35]. Where psychosocial items have been included in other tools, selection was not empirically-based but reflected service intentions to identify and address adjunct issues such as intimate partner violence [36].

Our findings also suggest that different items may indicate different sexual risk experiences. E.g. results indicate that ‘not cohabiting with a partner’ is likely to perform better than ‘drug use ever’ in identifying women experiencing multiple partnerships, while specific identification of FSNC would rest on being older and not living with both parents until the age of 14 years. Finally, the symmetrical treatment of both psychosocial and socio-demographic variables in our analysis allowed us to examine associations between sexual risk and socio-demographic factors (namely age group and socio-economic status) while controlling for psychosocial factors. The findings indicate that these associations are not fully explained by psychosocial factors, and that socio-demographic questions should be combined with psychosocial questions in the CPR under development.

### Limitations

This analysis was limited by the variables available in the Natsal-3 dataset and by the prevalence of some of those variables given the size of the Natsal-3 sample. Hence non-significant findings may reflect a lack of statistical power. Similarly, low prevalence of unplanned pregnancy and of STI diagnoses in the population of interest precluded analysis of these outcomes. As the analysis focuses on women heterosexually active in the previous year, so our findings may not apply to women who were not sexually active in the last year, or who had sex exclusively with women (WSEW) in the last year. Nonetheless a large proportion of WSEW also have sex with men [38].

Unsurprisingly (as 2PPNC is a sub-category of 2PP) greater similarities were seen in the associations for these two overlapping variables. In contrast to 2PP and 2PPNC, FSNC represents only a proxy measure of risk behaviour. I.e., as it concerns only one episode of intercourse which may be non-recent so it cannot be considered a key risk factor for STI acquisition or UP in its own right. It is also important to note that, for some respondents, non-use of condoms may represent attempts to conceive, which is nonetheless considered a sexual risk behaviour for STIs. Natsal-3 was not designed to enable assessment of *prospective* unplanned pregnancy risk (i.e. lack of effective contraceptive use in those not wishing to become pregnant) and consequently this limited our analysis.

Education is commonly investigated as a proxy measure of socio-economic status. However it may carry psychosocial dimensions with regard to factors such as belonging, purpose, and social cohesion. However, none of the available education variables were suitable for use across all age groups. This reflects a common problem in such studies- that age is a major confounder of both duration of education and qualification attainment. Variables concerning non-volitional sex were also excluded from the analysis, as we anticipated that these questions would be unacceptable in a Primary Care- based assessment, so that measuring and adjusting for them in this analysis was of no practical benefit. This pragmatic approach to adjustment reflects the overarching purpose of this study in developing a CPR for primary care use. This stands in contrast to a conventional complex survey methodology approach - in which all factors likely to confound associations are adjusted for, to achieve a more accurate picture of how an exposure and outcome are independently associated.

Although a conservative approach was undertaken towards aggregation of response categories, wide confidence intervals may have led to aggregation of distinct categories with loss of data sensitivity as a result. Nonetheless, most of the substance use variables were associated with sexual risk outcomes, while very few of the relationship quality variables were; this topic-based pattern suggests that aggregation-based insensitivity was not likely to be responsible for the overall patterns of association.

## Conclusions

This study indicates that there are a number of variables which are worthy of further investigation for use in a Clinical Prediction Rule to identify women experiencing sexual risk in primary care settings, and are suitable for self-completion. Certain socio-demographic and psychosocial variables which were associated with only one or two of the risk behaviours studied may also be useful in differentiating between those needing STI testing or CAS.

From this analysis we cannot draw conclusions about causation. Our working definition of ‘psychosocial’ may have incorporated variables whose association with sexual risk behaviours represent spurious associations - rather than being ‘the causes of the causes’ [39]. Nonetheless, factors such as binge drinking may constitute wider determinants of sexual health, prevention of which may reduce sexual (and other) morbidity. This is highlighted by England’s Sexual Health Improvement Framework, 2013 [40].

We also cannot assume that the performance of questions investigated in Natsal-3 will directly transfer to a clinical prediction rule administered in Primary Care. This is because of differences in purpose (research versus clinical practice) and delivery (random sampling versus clinical delivery). Further research is focusing on the performance of psychosocial and socio-demographic variables as CPR questions in clinical settings – using the variables found in this analysis and in systematic review of relevant literature [10]. This ongoing work by the authors is investigating the degree and type of sexual risk explained by these variables, and their acceptability and delivery as questions used in primary care assessment.

## Additional file

Additional file 1: Description of data: List of Natsal-3 variables initially considered for variable inclusion. (DOCX 20 kb)

## Abbreviations

2PP: 2 or more sexual partners in the last year
2PPNC: 2 or more sexual partners in the last year with whom a condom was not used
AOR: Adjusted odds ratio
BMI: Body mass index
CAS: Contraceptive advice and supply
CPR: Clinical prediction rule
CSA: Childhood sexual abuse
FSNC: Non-use of condoms at first sex with most recent partner
Natsal-3: National Survey of Sexual Attitudes and Lifestyles
STI: Sexually transmitted infection
UP: Unplanned pregnancy
