# Supplementary material for: Can psychosocial and socio-demographic questions help identify sexual risk among heterosexually-active women of reproductive age? Evidence from Britain’s third National Survey of Sexual Attitudes and Lifestyles (Natsal-3)
Source: BMC Public Health. 2017 Jan 4;17:5. doi: 10.1186/s12889-016-3918-8 (PMC5209946; doi:10.1186/s12889-016-3918-8)
Supplement: Additional file 1: — Description of data: List of Natsal-3 variables initially considered for variable inclusion. (DOCX 20 kb) [file 12889_2016_3918_MOESM1_ESM.docx]

**Additional File 1**

Listed here are the 42 Natsal-3 variables considered for inclusion in the model as psychosocial variables or as socio-demographic or other confounders (grouped by type). The variables which were then selected for possible model inclusion are underlined.

**Relationships**

- Relationship status (cohabiting with partner/stable relationship not cohabiting/not in a relationship but previously cohabited/not in a relationship and never cohabited)
- Marital status at present (married or civil partnership and living with partner/cohabiting but not married or civil partnership/previously married or civil partnership/never married or had civil partnership and not cohabiting)
- How happy are you in your relationship with your partner (not applicable/1/2/3/4/5/6/7)
- Share same sexual likes and dislikes as partner (not applicable/agree strongly/agree/neither agree nor disagree/disagree/strongly disagree)
- Share same level of interest in having sex as partner (not applicable/agree strongly/agree/neither agree nor disagree/disagree/strongly disagree)
- How long experiencing lack of interest in sex (not applicable/3-6 months/6-12 months/1-5 years/5+ years)
- Preference of frequency of sex (not applicable/to have sex much more often/to have sex a bit more often/about right/to have sex a bit less often/to have sex much less often)
- Feel distressed or worried by sex life (not applicable/agree strongly/agree/neither agree nor disagree/disagree/strongly disagree)
- One week or less between meeting most recent partner and first sex (yes/no)
- How easy is it to talk about sex with your most recent partner (for those reporting 2+ partners) (not applicable/ easy with spouse/regular partner but difficult with new partner/easy with new partner but difficult with previous partner/easy with any partner/difficult with any partner/sometimes easy, sometimes difficult)
- Where first met most recent partner (17 response options)
- Partner ethnicity (white/Asian British/black British/other)

**Sexual identity**

- Sexual identity (heterosexual/ not heterosexual)

**Religion**

- Belonging to a religion (yes/no)
- Importance of religion and religious beliefs now (very/ fairly/ not very/not important at all)
- Frequency of attendance to religious meetings (not applicable/at least once a month/less often but at least once a year/less often than once a year)

**Substance use**

- Smokes cigarettes nowadays (yes/no)
- Current smoker status (non/ex/light/heavy)
- How often have you had more than six units on one occasion? (less than weekly/at least weekly)
- How often have you had more than six units on one occasion in the last year? (never/less than monthly/monthly/weekly/daily or almost daily)
- Average alcohol consumption per week in units (0/1-7/8-14/15-21/>21)
- Average frequency of alcohol consumption in the last 12 months of 3 or more days a week (yes/no)
- Ever taken drugs (yes/no)
- Have you taken cannabis in the last 12 months (yes/no)
- Have you taken drugs other than cannabis in the last 12 months (yes/no)
- Have you taken cannabis in the last 4 weeks (yes/no)
- Have you taken drugs in the last year (yes/no)

**General health**

- Respondent’s opinion of own health as fair or better than fair (yes/no)
- Treatment for a medical condition in the last year (yes/no)
- Respondent weight in kgs (continuous)
- Backache lasting for 3+ months in the last year (yes/no)

**Mental health**

- Currently taking medication for depression (yes/no)
- Received treatment for depression in the last year (yes/no)
- Positive screen for current depression based on PHQ9-2 (yes/no)

**Formative experiences**

- First heterosexual intercourse<age 16 years (yes/no)
- Lived with both parents until the age of 14 (yes/no)
- Discussed sex with parents at age 14 (didn’t discuss with either parent/discussed with 1+ parent/ didn’t live with either parent continuously)

**Education**

- Passed any exams or got any qualifications (yes/no)
- Completed continuous full-time education at age 16 or less (yes/no)

**Respondent socio-demographics**

- Age group (16-24/25-34/35-44)
- Tenure (renting/not renting)
- Ethnicity (white/Asian British/black British/other)
